# Prostaglandins in Superovulation Induced Bovine Follicles During the Preovulatory Period and Early Corpus Luteum

**DOI:** 10.3389/fendo.2019.00467

**Published:** 2019-07-10

**Authors:** Bajram Berisha, Daniela Rodler, Dieter Schams, Fred Sinowatz, Michael W. Pfaffl

**Affiliations:** ^1^Department of Animal Biotechnology, Faculty of Agriculture and Veterinary, University of Prishtina, Pristina, Kosovo; ^2^Animal Physiology and Immunology Weihenstephan, Technical University of Munich, Munich, Germany; ^3^Department of Veterinary Sciences, Ludwig Maximilian University of Munich, Munich, Germany

**Keywords:** prostaglandins, steroids, gene regulation, ovarian function, follicle, ovulation, cow

## Abstract

The aim of this study was to characterize the regulation pattern of prostaglandin family members namely prostaglandin F2alpha (PTGF), prostaglandin E2 (PTGE), their receptors (PTGFR, PTGER2, PTGER4), cyclooxygenase 2 (COX-2), PTGF synthase (PTGFS), and PTGE synthase (PTGES) in the bovine follicles during preovulatory period and early corpus luteum (CL). Ovaries containing preovulatory follicles or CL were collected by transvaginal ovariectomy (*n* = 5 cows/group), and the follicles were classified: (I) before GnRH treatment; (II) 4 h after GnRH; (III) 10 h after GnRH; (IV) 20 h after GnRH; (V) 25 h after GnRH, and (VI) 60 h after GnRH (early CL). In these samples, the concentrations of progesterone (P4), estradiol (E2), PTGF and PTGE were investigated in the follicular fluid (FF) by validated EIA. Relative mRNA abundance of genes encoding for prostaglandin receptors (PTGFR, PTGER2, PTGER4), COX-2, PTGFS and PTGES were quantified by RT-qPCR. The localization of COX-2 and PTGES were investigated by established immunohistochemistry in fixed follicular and CL tissue samples. The high E2 concentration in the FF of the follicle group before GnRH treatment (495.8 ng/ml) and during luteinizing hormone (LH) surge (4 h after GnRH, 574.36 ng/ml), is followed by a significant (P<0.05) downregulation afterwards with the lowest level during ovulation (25 h after GnRH, 53.11 ng/ml). In contrast the concentration of P4 was very low before LH surge (50.64 mg/ml) followed by a significant upregulation (*P* < 0.05) during ovulation (537.18 ng/ml). The mRNA expression of COX-2 increased significantely (*P* < 0.05) 4 h after GnRH and again 20 h after GnRH, followed by a significant decrease (*P* < 0.05) after ovulation (early CL). The mRNA of PTGFS in follicles before GnRH was high followed by a continuous and significant downregulation (*P* < 0.05) afterwards. In contrast, PTGES mRNA abundance increased significantely (*P* < 0.05) in follicles 20 h after GnRH treatment and remained high afterwards. The mRNA abundance of PTGFR, PTGER2, and PTGER4 in follicles before GnRH was high, followed by a continuous and significant down regulation afterwards and significant increase (*P* < 0.05) only after ovulation (early CL). The low concentration of PTGF (0.04 ng/ml) and PTGE (0.15 ng/ml) in FF before GnRH, increased continuously in follicle groups before ovulation and displayed a further significant and dramatic increase (*P* < 0.05) around ovulation (101.01 ng/ml, respectively, 484.21 ng/ml). Immunohistochemically, the granulosa cells showed an intensive signal for COX-2 and PTGES in follicles during preovulation and in granulosa-luteal cells of the early CL. In conclusion, our results indicate that the examined bovine prostaglandin family members are involved in the local mechanisms regulating final follicle maturation and ovulation during the folliculo-luteal transition and CL formation.

## Introduction

The ovarian cycle in bovine is characterized by regularly repeating patterns of cellular proliferation, differentiation and transformation that accompanies follicular maturation and ovulation during the folliculo-luteal transition and corpus lutem (CL) formation and function (1–5). It is well-known that the ruminant reproductive function and especially the ovarian cycle is regulated through endocrine, as well as intraluteal (autocrine/paracrine) actions (6–9). The LH surge triggers a biochemical cascade that leads to the ovulation, resulting in development of the CL (10–12). Ovulation occurs as a result of a dynamic interaction between the luteinising hormone (LH) surge and local follicular factors including steroid hormones, extracellular matrix (ECM) proteases, prostaglandins, vasoactive peptides and growth factors in a time-dependent manner (1, 13–15). During these developments in the bovine ovary, steroid hormones and prostaglandins seem to be highly important regulatory mediators playing a central role in the regulation of the estrous cycle (16–22). Progesterone (P4) and estradiol (E2) steroid production of ovulatory follicles change dramatically during the preovulatory period, suggesting them to have an important role during ovulation (19, 23–25). The later stage of follicular development, ovulation and CL formation depends upon growth of new blood vessels (angiogenesis) and the establishment of a functional blood supply (12, 26, 27).

A recent finding demonstrates that steroid hormones and prostaglandins in addition to different angiogenic factors are required for angiogenesis and folliculo-luteal transition (28–33). The prostaglandins are of particular interest because of their endocrine as well as local effects within the ovarian tissue during different physiological stages (20, 34, 35). Intraluteal prostaglandin production is regulated by a variety of endocrine and autocrine/paracrine factors secreted by different immune cells, namely, macrophages, eosinophils, lymphocytes and monocytes (5, 8, 36). The production of prostaglandins from arachidonic acid is primarily governed by the rate-limiting enzymes cyclooxygenase (COX)-1 and COX-2 (16, 34). The downstream enzymes, PTGF synthase (PTGFS) and PTGE synthase (PTGES), catalyze the conversion of prostaglandin H2 precursors to prostaglandin F2alpha (PTGF) and prostaglandin E2 (PTGE) respectively, (14, 20). PTGF has the highest affinity for the specific receptor (PTGFR), and PTGE may interact with at least four receptor subtypes (PTGER1—PTGER4) and initiate biological signaling pathways (21, 37–39).

The steroid hormones and prostaglandins were shown to regulate ovarian cycle in cattle, but the examination of these factors during final follicle regulation, ovulation and CL formation, has not been thoroughly elucidated to date. Therefore, we tested the hypothesis if the preovulatory LH surge may affect COX-2, prostaglandin synathases (PTGFS and PTGES), prostaglandin ligands (PTGF and PTGE) and their receptors (PTGFR, PTGER2, and PTGER4), which may have further effects to the folliculo-luteal transition and CL formation in the cow. With the present study, we aim to evaluate the expression pattern and localization of earlier mentioned prostaglandin family members in time-defined follicle classes before (control) and after the application of GnRH and after ovulation (early CL) in the cow.

## Materials and Methods

### Animals, Procedure of Superovulation and Collection of Ovaries

**Figure 1:**
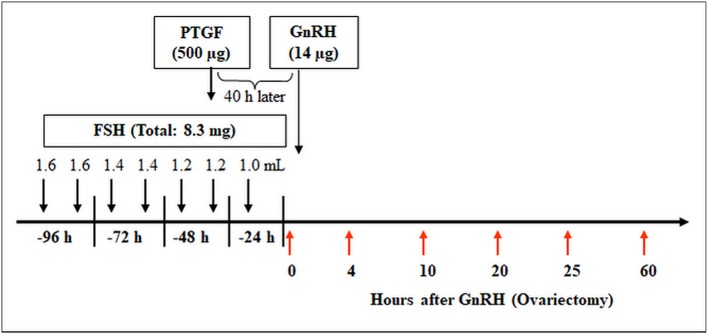
Time schedule of the treatment for multiple ovulation and ovary collection in cows. Ovaries containing preovulatory follicles or new CL were collected at (I) 0 h, (II) 4 h, (III) 10 h, (IV) 20 h, (V) 25 h (follicles), and (VI) 60 h (early CL, Day 2–3) relative to injection of GnRH to induce an luteinizing hormone (LH) surge (*n* = 5 cows/group).

The animal trail was approved by the animal ethics committee located at the government of Upper Bavaria (reference number 211-2531.3-33/96). The study was conducted on 30 German Fleckvieh cows and the superovulation procedure was conducted as described by Berisha et al. (40). For confirmation of LH surge, blood samples were collected from the jugular vein at −24 h, −12 h, −1 h, and 0 h before and 3 h and 12 h after GnRH application (27). The bovine ovaries (containing preovulatory follicles or early CL) were collected at (I) 0 h, (II) 4 h, (III) 10 h, (IV) 20 h, (V) 25 h (for follicle collection) and (VI) 60 h (for early CL collection) relative to injection of GnRH (n = 5 cow/ group) as described by Berisha et al. (27). The schematic time schedule of the superovulatory treatment and ovary collection is shown in Figure 1.

### Collection, Classification and Preparation of Preovulatory Follicles and Early CL

Only follicles which appeared healthy (i.e., well-vascularised and having transparent follicular wall and fluid) and whose diameter were >10 mm were collected. The number of follicles per ovary varied between 8 and 20. Follicular fluid (FF) was aspirated from single follicles, and then the follicles tissue (theca interna and granulose cells) aliquots were stored at −80°C until extraction for RNA. The FF (1.0–1.5 ml) was stored from single follicles at −20°C until determination of P4, E2, PTGF, and PTGE (40). The follicles, after aspiration of FF and injection of fixative (27) and pieces of CL tissue were fixed for immunohistochemical analysis of COX-2 and PTGES (22).

### Hormone Determinations

The concentrations of P4, E2, PTGF, and PTGE were determined in the FF with an enzyme immunoassay (EIA) using the second antibody technique as described by our lab and reviewed by Berisha et al. (40). The concentration of progesterone in blood plasma was measured using EIA technique as described by Berisha et al. (41).

### Total RNA Extraction and Quality Determination

Total RNA from 200 mg follicles and CL (deep frozen by −80°C) were extracted with peqGOLD TriFAst (PeqLab, Erlangen, Germany) according to the manufacturer's instructions and described by Berisha et al. (40). For DNA digestion the DNA-free kit (Ambion, Austin, USA) was used. Total RNA was dissolved in RNAse-free water and spectroscopically quantified at 260 nm. The purity of isolated RNA was verified by optical density (OD) absorption ratio OD260 nm/OD280 nm between 1.8 and 2.0.

The RNA integrity was measured with the Agilent 2100 bioanalyzer (Agilent Technologies, Deutschland Gmbh, Waldbronn, Germany) in conjunction with the RNA 6000 Nano Assay according to the manufacturer's instructions. The Bioanalyzer 2100 enables the standardization of total RNA quality control for quantitative downstream applications (42). The automatically calculated RNA Integrity Number (RIN) allows classification of total RNA based on a numbering system from 1 to 10, with 1 being the most degraded profile and 10 being the most intact (43). Herein integer total RNA with RIN values of 7–8 were achieved over all tissue extractions.

### RNA Reverse Transcription and Real-Time PCR

Constant amounts of 1 μg of total RNA were reverse-transcribed to cDNA using the following master mix: 26 μl RNAse-free water, 12 μl 5 × Buffer (Promega, Mannheim, Germany), 3 μl Random Primers (50 μM) (Invitrogen, Carlsbad, Germany), 3 μl dNTPs (10 mM) (Fermentas, St. Leon-Rot, Germany) and 200 U of M-MLV Reverse Transcriptase (Promega, Mannheim, Germany) according to the manufacturer's instructions. A master mix of the reaction components was prepared according to Berisha et al. (22). The following Real-Time PCR protocol was employed for all investigated factors: denaturation for 10 min at 95°C, 40 cycles of a three segmented amplification and quantification program (denaturation for 10 s at 95°C, annealing for 10 s at 60°C, elongation for 15 s at 72°C), a melting step by slow heating from 60 to 99°C with a rate of 0.5°C/s and continuous fluorescence measurement, and a final cooling down to 40°C. Data were analyzed using Rotor-Gene 3000 software (Corbett Research version 5.03). The relative mRNA abundance of each target gene were calculated using the “comparative quantification” method (Corbett Reasearch). The changes in mRNA expression of examined target genes were assayed by normalization to the stable expressed and internal UBQ control gene. In order to obtain the CT (cycle threshold) difference the data were analyzed using the well-established ΔΔCT method described by Livak and Schmittgen (44). Thereby ΔCT was not subtracted from a non-treated control group, which does not exist in this study, but from the constant number 40, so that a high “40-ΔCT” value indicated a high-gene expression level and vice versa. This results in directly comparable relative expression values between the examined follicle classes before and after the application of GnRH and after ovulation (early CL) in the cow.

### Immunohistochemistry of COX-2 and PTGES

Paraffin-embedded mature follicles and CL tissue (fixed in Bouin's fluid for 24 h) were cut into 5-μm serial sections and collected on amino-propyltriethoxysilane coated slides (SupraFrost Ultra Plus; Menzel-Gläser, Braunschweig, Germany). Paraffin sections were dewaxed and then washed three times for 5 min with PBS at pH 7.4. The sections were incubated with polyclonal primary antibodies to PTGES (ab62050; diluted 1:300, host rabbit; Abcam, Cambridge, UK; secondary antibody: pig anti-rabbit IgG (F(ab′)2), diluted 1:300) and with polyclonal primary antibodies to COX-2 (ab2367; diluted 1:400, host goat; Abcam; secondary antibody: rabbit-anti-goat IgG (F(ab′)2), diluted 1:300) at 6°C overnight. Endogenous peroxidase activity was blocked with 7.5% H_2_O_2_ (diluted in distilled water) at room temperature for 10 min.

Nonspecific antibody binding was blocked with Dako protein block serum-free (Dako Deutschland GmbH; Hamburg, Germany) for 10 min. The sections were incubated with the primary antibodies at 6°C overnight. Localization of the antigen was achieved using the avidin–biotin-complex technique. The appropriate biotinylated secondary antibodies were incubated with the sections for 16 h at room temperature. Subsequently, treatment with Strept-ABComplex-HRP (Dako Deutschland GmbH) was performed for 30 min at room temperature, and treatment with 1 mg/ml 3,3-diaminobenzidinetetrahydrochloride (BIOTREND Chemikalien GmbH; Cologne, Germany) was performed for 5 min. All incubations were performed in a humidified chamber.

Sections were slightly counterstained with haematoxylin (20 s), dehydrated and mounted with the Eukitt quick hardening mounting medium for microscopy (Fluka Analytical; Sigma-Aldrich Laborchemikalien GmbH, Seelze, Germany). Negative controls were performed by incubating with the 3,3-diaminobenzidine reagent alone to exclude the possibility of detecting the non-suppressed endogenous peroxidase activity. A lack of detectable staining in the negative controls demonstrated that the reactions were specific. The images were captured with a Leica Labo-Lux microscope equipped with a Zeiss Axiocam camera (Zeiss, Munich, Germany). As positive controls, ovarian tissues from quails (Coturnix japonica) and cats (Felis silvestris) of proven immunoreactivity were used (45). The reaction intensities were marked as weak (+), distinct (++), and strong (+ + +).

### Statistical Analysis

The statistical significance of differences in hormone concentration in FF and mRNA expressions in follicle and CL tissue of the examined factors was assessed by one way ANOVA followed by the Holm Sidak as a multiple comparison test. Data, which failed the normality or equal variance test, were tested by one way ANOVA on ranks followed by the Kruskal-Wallis test (Sigma Stat 3.0). All experimental data are shown as means±SEM (*n* = 8–12). The differences were considered significant if *P* < 0.05.

## Results

### Concentration of E2, P4 and PTGE and PTGF in FF During Preovulation

We analyzed the concentration of E2, P4, PTGF, and PTGE in FF for a better characterization of follicle groups before and after GnRH application and during ovulation (Table 1).

**Table 1:** Concentration of Prostaglandin F2alpha (PTGF), Prostaglandin E2 (PTGE), Estradiol (E2), and Progesterone (P4) in follicular fluid (FF) of preovulatory follicles collected at (I) 0 h, (II) 4 h, (III) 10 h, (IV) 20 h, and (V) 25 h relative to injection of GnRH to induce an LH surge.

**Follicle groups (hours after GnRH administration)**					
**Hormones in FF (ng/ml)**	**Group I (0 h)**	**Group II (4 h)**	**Group III (10 h)**	**Group IV (20 h)**	**Group V (25 h)**
PTGF	0.04 ± 0.1^b^	1.50 ± 0.5^b^	1.18 ± 0.26^b^	36.25 ± 7.13^ab^	101.01 ± 33.42^a^
PTGE	0.15 ± 0.03^c^	1.21 ± 0.26^c^	3.60 ± 0.63^bc^	15.16 ± 6.07^b^	484.21 ± 109.27^a^
E2	495.8 ± 80.09^a^	574.36 ± 109.7^a^	178.96 ± 29.06^b^	59.25 ± 8.4^b^	53.11 ± 7.28^b^
P4	50.64 ± 11.49^b^	186.41 ± 45.60^b^	130.63 ± 20.17^b^	203.78 ± 32.82^b^	537.18 ± 81.26^a^

*Results are presented as the mean ± SEM (ng/ml FF, n = 8–12 follicle/group from 5 animals). Different superscripts denote significantly different values (P <0.05)*.

The high E2 concentration in the FF of the follicle group before GnRH treatment (495.8 ng/ml) and during luteinizing hormone (LH) surge (4 h after GnRH, 574.36 ng/ml), is followed by a significant (*P* < 0.05) downregulation afterwards with the lowest level during ovulation (25 h after GnRH, 53.11 ng/ml). In contrast the concentration of P4 was very low before LH surge (50.64 mg/ml) followed by a significant upregulation (*P* < 0.05) during ovulation (537.18 ng/ml).

The low concentration of PTGF (0.04 ng/ml) and PTGE (0.15 ng/ml) in FF before GnRH, increased continuously in follicle groups before ovulation and displayed a further significant and dramatic increase (P <0.05) around ovulation (101.01 ng/ml respectively 484.21 ng/ml).

### Confirmation of Primer Specificity and Sequence Analysis

The mRNA expression was quantified by the reverse transcription quantitative polymerase chain reaction (RT-qPCR), as described in detail in our previous study (46). Amplified RT-qPCR products were separated on agarose gel electrophoresis for length verification, and for sequence confirmation additionally sequenced by a commercial provider (TopLab, Munich, Germany). All amplified RT-qPCR products showed 100% homology to the known bovine gene sequence published in NCBI GenBank (complete sequences are not shown herein). The primer sequences and expected PCR product length are shown in Table 2.

**Table 2:** Primer sequences for investigates genes, respective RT-qPCR product length, and appropriate reference.

**Target**	Sequence of nucleotide fragment^*^	**Size (bp)**	**References**
UBQ	For 5′-AGATCCAGGATAAGGAAGGCAT-3′ Rev 5′-GCTCCACCTCCAGGGTGATT-3′	189	(30)
GAPDH	For 5′-GTCTTCACTACCATGGAGAAGG-3′ Rev 5′-TCATGGATGACCTTGGCCAG-3′	197	(30)
COX-2	For 5′-CTCTTCCTCCTGTGCCTGAT-3′ Rev 5′-GACTCATAGAAACTGACACCCTC-3′	359	(30)
PTGFS	For 5′-ACCTGGACCTCTACCTCATCCA-3′ Rev 5′-TCCTCATCCAATGGGAAGAAGT-3′	100	(22)
PTGES	For 5′-GCGCGCTGCTGGTCATCAAA-3′ Rev 5′-GTGTAGGCCAGGGAGCGGGT-3′	334	(22)
PTGFR	For 5′-TCAGCCCTCACCCAGATAGT-3′ Rev 5′-GGCCATTTCACTGTTCAGGT-3′	167	(22)
PTGER2	For 5′-CTACTTTGCCTTTTCCATGACC-3′ Rev 5′-GATGAAGCACCACGTCCC-3′	210	(22)
PTGER4	For 5′-CGATGAGTATTGAGCGCTACC-3′ Rev 5′-AGCCCGCATACATGTAGGAG-3′	220	(22)

* *For, forwards; Rev, reverse*.

### Relative mRNA Abundance

**Figure 2:**
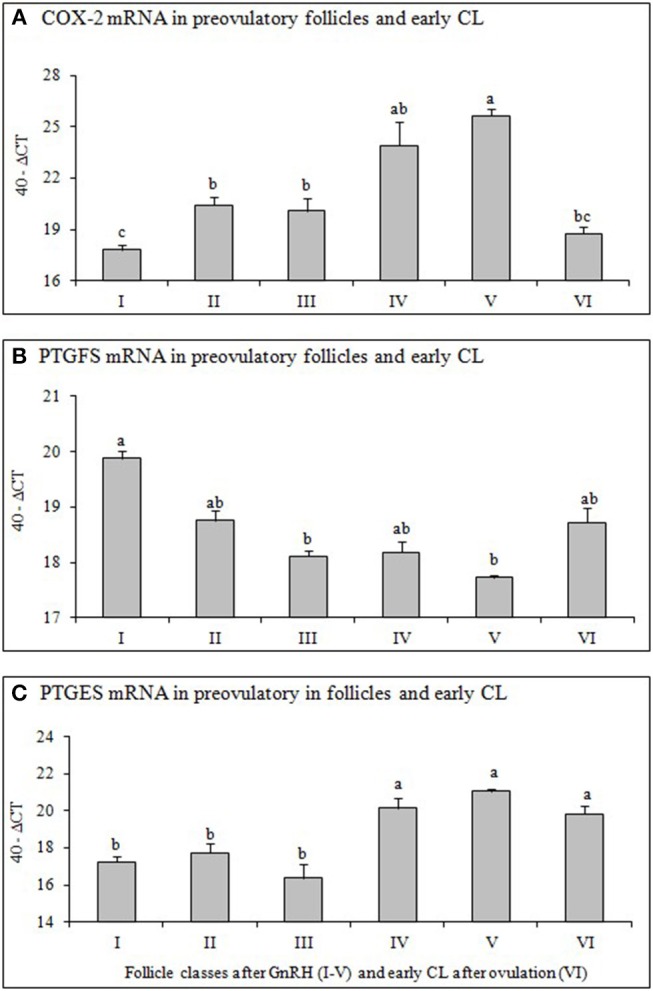
Changes of mRNA for **(A)** Cyclooxygenase 2 (COX-2), **(B)** Prostaglandin F2alpha synthase (PTGFS) and **(C)** Prostaglandin E2 synthase (PTGFS) in preovulatory follicles collected at (I) 0 h, (II) 4 h, (III) 10 h, (IV) 20 h, (V) 25 h (follicles), and (VI) 60 h (new CL, Day 2–3) relative to injection of GnRH to induce an LH surge (5 follicles or CL per group from 5 animals). Different superscripts denote statistically different values (*P* < 0.05).

**Figure 3:**
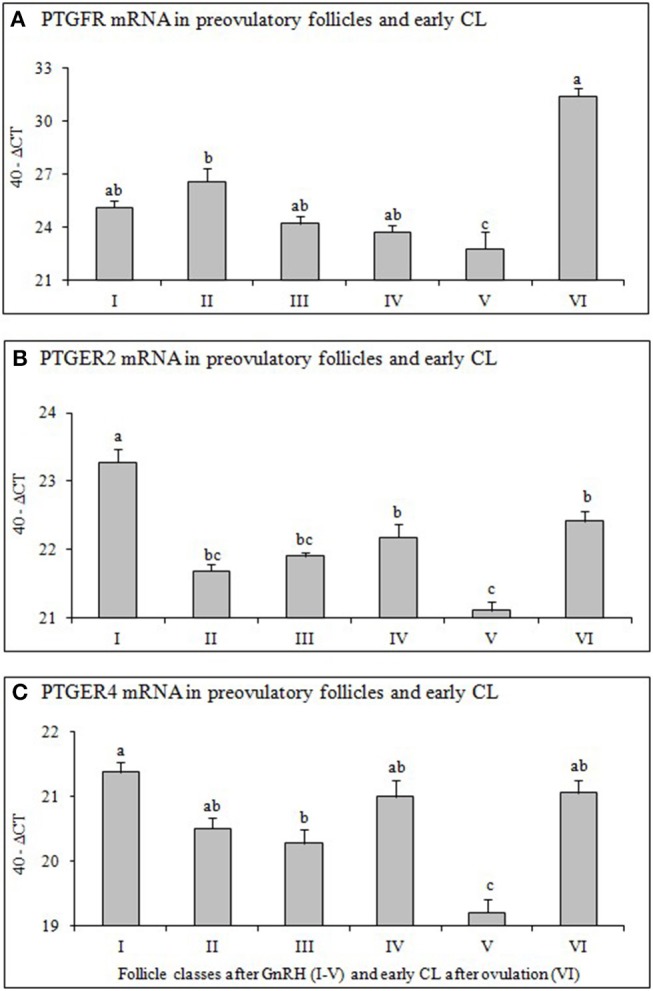
Expression of mRNA for **(A)** Prostaglandin F2alpha receptor (PTGFR), **(B)** Prostaglandin E2 receptor 2 (PTGER2), and **(C)** Prostaglandin E2 receptor 4 (PTGER4) in preovulatory follicles collected at (I) 0 h, (II) 4 h, (III) 10 h, (IV) 20 h, (V) 25 h (follicles), and (VI) 60 h (new CL, Day 2–3) relative to injection of GnRH to induce an LH surge (5 follicles or CL/group from 5 animals). Different superscripts denote statistically different values (*P* < 0.05).

To evaluate equal quantity and quality of the preceding RT reaction in each sample, the housekeeping genes ubiquitin (UBQ) and glyceraldehyde-3-phosphate dehydrogenase (GAPDH) were examined in all samples. As both housekeeping genes were constantly expressed in all samples we choose UBQ as normalizer. The results of mRNA expression of examined factors (Figures 2, 3) are presented as changes (40-ΔCT ± SEM from 6 follicles or CL per group) in the target gene expression, normalized to UBQ as described by Berisha et al. (22).

### Relative mRNA Expression of COX-2, PTGES and PTGFS in Preovulatory Follicles and Early CL

The mRNA expression of COX-2 (Figure 2A) increased significantely (*P* < 0.05) 4 h after GnRH and again 20 h after GnRH, followed by a significant decrease (*P* < 0.05) after ovulation (early CL). The mRNA of PTGFS (Figure 2B) in follicles before GnRH was high followed by a continuous and significant downregulation (*P* < 0.05) afterwards. In contrast, PTGES mRNA abundance increased significantely (*P* < 0.05) in follicles 20 h after GnRH treatment and remained high afterwards (Figure 2C).

### Relative mRNA Expression of PTGFR, PTGER2 and PTGER4 in Preovulatory Follicles and Early CL

The mRNA abundance of PTGFR (Figure 3A), PTGER2 (Figure 3B), and PTGER4 (Figure 3C) in follicles before GnRH was high, followed by a continuous and significant down regulation afterwards and significant (*P* < 0.05) increase only after ovulation (early CL).

### Immunohistochemical Localization of COX-2 in Preovulatory Follicles and Early CL

**Figure 4:**
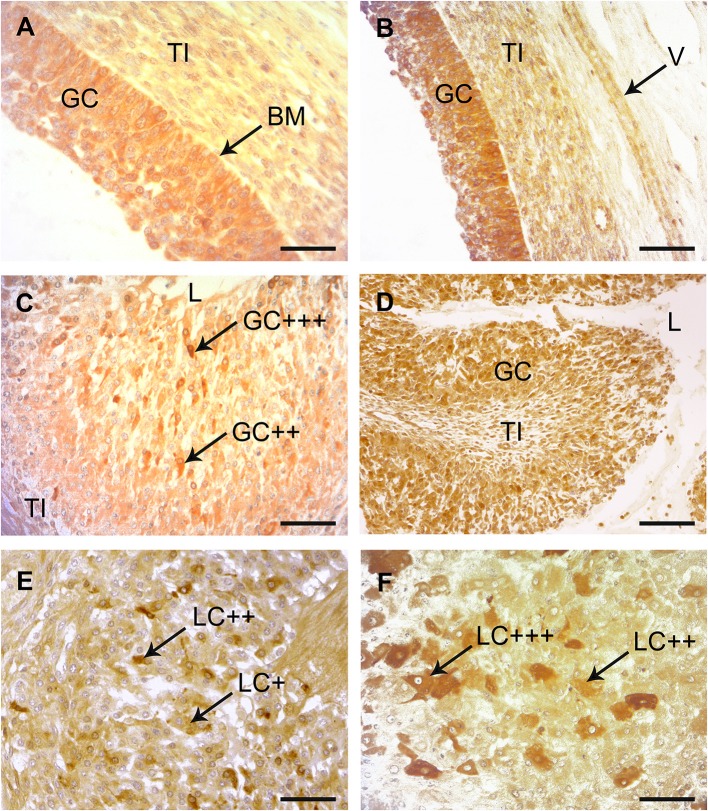
Immunohistochemical localization of COX-2 and PTGES in bovine preovulatory follicles and early corpus lutem (CL). **(A)** Immunohistochemical localization of COX-2 in a mature preovulatory follicle. A distinct to strong immunostaining was found throughout the granulosa cells (GC) with increaded intensity adjacent to the basement membrane (BM). The theca interna (TI) was also weakly stained. SB = 75 μm. **(B)** Immunohistochemical localization of PTGES in a mature preovulatory follicle. A distinct to strong immunostaining is found in the cytoplasma of the GC. Similar to the result with COX-2, the basal cells show stronger reactions than the luminal cells. The TI and the endothelium of this longitudinal cut vessel (V) show slight positive reactions. SB = 150 μm. **(C)** Immunohistochemical localization of COX-2 in the CL at days 1–2. A subpopulation of the folded membrana granulosa (GC) surrounding the remaining lumen (L) of the former antrum of the ovulated follicle display distinct (++) to strong (+ + +) positive immunostaining with the COX-2 antibody. TI = theca interna; SB = 100 μm. **(D)** Immunohistochemical localization of PTGES in the CL at days 1–2. Many of the former GC display distinct immunostaining. L = lumen, TI = theca interna; SB = 200 μm. **(E)** Immunohistochemical localization of COX-2 in the CL at days 3–4. At this magnification many granulosa-luteal cells (LC) with distinct (++) immunostaining alternate with negative or only weakly stained cells (+). SB = 150 μm. **(F)** Immunohistochemical localization of PTGES in the CL at days 3–4. Several of the granulosa-luteal cells (LC) show strong immunostaining (+ + +) with PTGES antibodies, whereas the rest of this cell population display only distinct to moderate immunopositivity (++). SB = 75 μm.

The multilayered epithelium of follicles at the time of ovulation (follicle diameter 18- 25 mm) displayed a clear signal for COX-2 (Figure 4A). COX-2 was expressed in the cytoplasm of more than 90% of the high prismatic basal cells located at the top of the basal membrane (BM). A slight staining was also seen in the theca interna surrounding the follicular epithelium. On days 1–2 after ovulation a strong signal for COX-2 could be noted in a subpopulation of cells located in the apical half of the folded membrana granulosa around the former antrum (Figure 4C). They were surrounded by other granulosa cells (GC), which showed only a weak signal. On days 3–4 scattered and distinctly COX-2-positive progesterone producing granulosa-luteal cells (LC) occurred in the developing corpus luteum (Figure 4E).

### Immunohistochemical Localization of PTGES in Preovulatory Follicles and Early CL

A distinct to strong immunoreactivity for PTGES was found throughout the follicular epithelium before ovulation (Figure 4B). Most cells of the theca interna (TI) displayed only weak immunoreactivity for this enzyme and the stromal cells of the theca were almost negative. On days 1–2 after ovulation, granulosa cells (GC) of the developing CL showed distinct signal with the PTGES antibody (±). The signal intensity of the granulosa-luteal cells (LC) increased in the CL at days 3–4 (Figure 4F).

## Discussion

Recent studies have demonstrated the important role of steroid hormones and prostaglandins during follicle development, ovulation and CL formation in different species and various study models (8, 22, 31, 47, 48). Our present study demonstrates the expression pattern of steroid hormones (E2 and P4) and prostaglandin family members (COX-2, PTGFS, PTGES, PTGF, PTGE, and their receptors) in different timely defined follicle classes before and after GnRH application and after ovulation (early CL) in the cow. We have shown in our previous studies (40, 49) that superovulated follicles after GnRH application are comparable to natural ovulation in the cow. We demonstrated in addition that also the time interval between the LH surge and ovulation is quite comparable between induced ovulation (40) and spontaneous ovulation in cows (49).

The LH surge initiates a series of biochemical events in ovary, such as upregulation of steroids, prostaglandins, ECM proteases and many locally produced growth and angiogenic factors to complement gonadotropins action in process of ovulation and CL formation (8, 13, 27, 50–52).

It is well-known that P4 and E2 steroid production of preovulatory follicles change dramatically during the periovulatory period, suggesting them to play an important role during final follicle development and ovulation (11, 14, 23–25, 53, 54). In addition our previous studies demonstrated clear evidence for steroids as local regulators of follicular and luteal activity (4, 19). *In vivo* and *in vitro* studies demonstrated production and localization of P4 and E2 and their specific receptors in both granulosa and theca cells of perovulatory follicles (11, 14, 23–25). The E2 concentration in the FF of our study (Table 2) was high in the follicle group before and during LH surge, followed by a significant downregulation afterwards. In contrast the concentration of P4 was very low before LH surge followed by a significant upregulation during LH surge, with maximal level during ovulation (Table 2). Our present data agree with results of Fortune et al. (11) suggesting that increase in follicular P4 production and associated decreases in E2 concentration in FF prior ovulation, reflect transition from a follicular to a luteal steroidogenic profile of cells.

The results of our present study clearly demonstrate the expression pattern of examined prostaglandin family members, which depends on the developmental stage of the follicles before and after LH surge and after ovulation (early CL) in the bovine ovary. The multilayered epithelium of mature follicles shows a distinct to strong signal for COX-2 and PTGES, whereas the theca interna is only weakly positive (Figure 4). At day 1–2 after ovulation, a subpopulation of GC of the former follicle epithelium is distinctly immunopositive for COX-2 and PTGES. According to Fortune et al. (11), the major source of prostaglandins is believed to be GC of the preovulatory follicle. At day 3–4 large (granulosa luteal cells) and small (theca luteal cells) can be discerned. A subpopulation of the large luteal cells is distinctly immunopositive for both enzymes. As COX-2 and PTGES are key enzymes for the synthesis of prostaglandins, their similar expression pattern makes sense.

It is well-known that LH surge and ovulation process influence production of COX, prostaglandin synthases (PTGFS and PTGES), prostaglandin ligands (PTGF and PTGE) but also regulates expression of prostaglandin receptors (PTGFR, PTGER2, and PTGE4). In our present study the concentration of PTGF and PTGE in FF (Table 2) was low prior to LH surge (before GnRH application), increased continuously and significantly 20h after GnRH and further increase around ovulation (25 h after GnRH). The rapid increase of PTGF and especially PTGE in preovulatory follicles close to ovulation is in accordance with previous studies in bovine and other mammals (55–57). The data of Fortune et al. (11) supported the hypothesis that prostaglandins, especially PTGE, can stimulate P4 secretion by both follicular cell types and suggest a positive feedback relationship between P4 and the prostaglandins. In addition, Bridges and Fortune (21) demonstrated that both theca and GC of preovulatory follicles are targets for both the PTGF and the PTGE produced by the GC. The results of some earlier studies showed clearly that ovulation process is dependent from follicular prostaglandin production also in cows (23, 47, 53–59). LH surge has been reported to induce enzymes modulating prostaglandin production and specific prostaglandin receptors in the preovulatory follicles (21, 33, 39, 60).

The upregulation of COX-2 and PTGES expression in our study was followed by a dramatic increase of PTGF and PTGE in the FF starting 20 h after GnRH application (Table 2). The preovulatory increases of prostaglandin concentration in FF have shown to be necessary component of the ovulation in several species (11, 33, 57, 59–61). Some earliest studies in different species suggested both PTGF and PTGE to be important to the ovulatory process (59, 62). However, the recent studies in different mammalian species clearly demonstrated that PTGE is more essential for ovulation and CL formation (14, 39, 47). Based on these findings, it is widely accepted that PGE2 is an essential paracrine mediator of the LH surge in mammalian (39, 57). The concentration of PTGE during ovulation in our study (Table 2) was much higher (484.21 ng/ml) than PTGF concentration (101.01 ng/ml). The marked increase of PTGF and especially PTGE in response to LH surge agrees with observation of other authors made in cow (23, 33, 59, 63).

The final follicle growth and ovulation process involves intense interactions between endothelial, steroidogenic and migrating immune cells, leading to the folliculo-luteal cell transition, ECM remodeling and further angiogenesis in the developing CL (9). Previous studies demonstrated the effects of LH surge in remodeling of ECM by regulation of diverse proteases of the plasminogen activators (PA) and the matrix metalloproteinase (MMP) enzyme systems in follicles during ovulation process and CL formation in cow (64–69). In addition Fortune et al. (11) demonstrated the effect of LH, P4 and prostaglandins in regulation of ADAMTS proteases (A Disintegrin And Metalloproteinase with Thrombo Spondin motifs), suggesting an important role of these factors in remodeling the preovulatory follicle during ovulation and angiogenesis. This study as well as others demonstrated that prostaglandins act via multiple receptors to regulate follicle-luteal transition and CL formation (22, 37–39, 57).

Not just the upregulation, but also the shift in the localization of different factors after LH surge suggests them to have an important role during the follicle-luteal transition. In our previous study in the same samples we have shown a distinct change in localization of FGF2 in the bovine follicles from the theca cell compartments (cytoplasm of endothelial cells) to the GC initiated by the LH surge. We suggested that nuclear FGF2 localization may be important for GC survival until ovulation and for a transition of GC to luteal cells in early CL (40). Examples of spatial differences are obvious for FGF2 (28) and, in particular, FGF7, for which the ligand is localized in the theca tissue and its receptor is localized mainly in GC (70). However, the aim of the present study was to analyze the regulation of the factors examined in whole follicle tissue (without GC and TI separation) in order to compare the expression between whole follicles tissue (GC and TI) and CL during follicle–luteal transition.

The significant upregulation of all prostaglandin receptors just after ovulation (early CL) in our study (Figure 3) demonstrates the important role of prostaglandins they play in the ovulation process, luteinisation and CL formation. Our previous study (22) demonstrated that PTGF and PTGE are involved in the local (autocrine/paracrine) mechanisms regulating CL formation and function. In early CL, the PTGE is known as a luteotropic factor, as it stimulates *in vitro* P4 production by bovine luteal steroidogenic cells (71, 72). In addition, PTGF stimulates P4 production, as well as PTGE, by cultured luteal cells (16, 73–75). Furthermore, PTGF and PTGE can suppress the apoptosis of steroidogenic and endothelial cells in bovine CL (72). In addition, our previous *in vivo* studies demonstrated that PTGF stimulates itself and PTGE in bovine CL (76, 77).

Moreover, in the early CL, prostaglandins regulate the expression of angiogenic factors in a luteal stage-dependent manner (9, 12, 35, 78). High expression and tissue levels of angiogenic factors during the early luteal stage (PTGF-refractory days) suggest a survival function for both endothelial cells of the capillaries and steroidogenic cells of the CL (12, 79). In our recent study we assume that LH modulates prostaglandin production in the bovine CL by stimulating the expressions of COX-2, prostaglandin synthases and angiogenic factors and that these actions help to maintain CL function during the early luteal phase (22). Moreover, intraluteal prostaglandins in the early CL may have different other functions, such as cellular transition and differentiation, blood flow regulation and intercellular communication (34, 80).

In conclusion, our results indicate that the LH surge upregulated prostaglandin family members in the follicle before ovulation, support the potential role of these system components in GC and TI tissue remodeling during ovulation process. The further upregulation of prostaglandin family members after ovulation (early CL) suggests that they play an important role in follicle-luteal transition and CL formation in the cow.

## Data Availability

All datasets generated for this study are included in the manuscript and/or the supplementary files.

## Ethics Statement

The experimental protocol was approved by the institutional care and use committee (AZ 211-2531.3-33/96).

## Author Contributions

BB and DS: designed and executed all the experiments. BB: wrote the manuscript. MP: qRT-PCR and data analysis. FS and DR: immunohistochemistry and data analysis. All the authors edited the manuscript.

### Conflict of Interest Statement

The authors declare that the research was conducted in the absence of any commercial or financial relationships that could be construed as a potential conflict of interest.
